# Structure of the Full-Length Major Pilin from *Streptococcus pneumoniae*: Implications for Isopeptide Bond Formation in Gram-Positive Bacterial Pili

**DOI:** 10.1371/journal.pone.0022095

**Published:** 2011-07-08

**Authors:** Neil G. Paterson, Edward N. Baker

**Affiliations:** Maurice Wilkins Centre for Molecular Biodiscovery and School of Biological Sciences, University of Auckland, Auckland, New Zealand; University of Canterbury, New Zealand

## Abstract

The surface of the pneumococcal cell is adorned with virulence factors including pili. The major pilin RrgB, which forms the pilus shaft on pathogenic *Streptococcus pneumoniae*, comprises four immunoglobulin (Ig)-like domains, each with a common CnaB topology. The three C-terminal domains are each stabilized by internal Lys-Asn isopeptide bonds, formed autocatalytically with the aid of an essential Glu residue. The structure and orientation of the crucial N-terminal domain, which provides the covalent linkage to the next pilin subunit in the shaft, however, remain incompletely characterised. We report the crystal structure of full length RrgB, solved by X-ray crystallography at 2.8 Å resolution. The N-terminal (D1) domain makes few contacts with the rest of the RrgB structure, and has higher B-factors. This may explain why D1 is readily lost by proteolysis, as are the N-terminal domains of many major pilins. D1 is also found to have a triad of Lys, Asn and Glu residues in the same topological positions as in the other domains, yet mass spectrometry and the crystal structure show that no internal isopeptide bond is formed. We show that this is because β-strand G of D1, which carries the Asn residue, diverges from β-strand A, carrying the Lys residue, such that these residues are too far apart for bond formation. Strand G also carries the YPKN motif that provides the essential Lys residue for the sortase-mediated intermolecular linkages along the pilus shaft. Interaction with the sortase and formation of the intermolecular linkage could result in a change in the orientation of this strand, explaining why isopeptide bond formation in the N-terminal domains of some major pilins appears to take place only upon assembly of the pili.

## Introduction

*Streptococcus pneumoniae* (Pneumococcus) is a major human pathogen responsible for a several life-threatening conditions in both industrialized nations and developing countries [1], [2]. Like other pathogenic bacteria, the surface of the pneumococcal cell is decorated with a variety of virulence factors that mediate host adhesion and assist invasion and infection [3], [4], [5], [6]. One of the most prominent of these virulence factors is the bacterial pilus; a long, thin proteinaceous filament extending from the cell wall that aids bacterial colonisation in the nasopharynx through epithelial cell binding [7].

Pili from Gram-negative bacteria have been well characterised and are formed from bundles of non-covalently associated proteins. They perform a variety of roles including adherence, motility and transfer of genetic information [8]. In contrast, Gram-positive pili are formed from a single chain of covalently linked subunit proteins (pilins), usually comprising an adhesin at the distal tip, a major pilin that forms the polymer shaft and a minor pilin that mediates cell wall anchoring at the base [9], [10]. Assembly of such structures is orchestrated by enzymes called sortases [11]. These cysteine transpeptidase enzymes recognize a characteristic sequence motif, usually LPXTG or similar (where X is any amino acid), near the C-terminus of a substrate protein, cleave the Thr-Gly bond and ligate the new C-terminal Thr carboxyl group to an amino group of a suitable acceptor molecule, with the formation of an isopeptide bond. For pilus assembly, specialized sortases ligate the pilin subunits, joining the Thr carboxyl of one subunit to the ε-amino group of a lysine residue on the next [9], [11], [12]. Finally a housekeeping sortase joins the entire pilus to the cell wall.

Crystal structures of the pilin subunits utilized by several Gram-positive bacteria show that this modular mechanism of Gram-positive pilus assembly is also reflected in the individual subunits [13]. The major pilins that form the polymeric backbones of the pili from *S. pneumoniae* [14], *Streptococcus pyogenes* [15], *Streptococcus agalactiae* [16], *Corynebacterium diphtheriae* [13] and *Bacillus cereus* [17] are in each case constructed from multiple Ig-like domains; two in the case of *S. pyogenes* Spy0128, three for *S. agalactiae* GBS80 and *C. diphtheriae* SpaA and four for *B. cereus* BcpA and *S. pneumoniae* RrgB. Similar Ig-like domains are used to make up the basal pilins [18] and the C-terminal portions of the adhesins that sit at the pilus tip [19], [20].

A striking feature of these Gram-positive pili is the widespread use of internal covalent cross-links that provide great stability to the pilin subunits in which they are found [15], [21], [22]. These cross-links take the form of isopeptide bonds that are formed autocatalytically between Lys and Asn (or Asp) side chains, aided by a neighboring Glu or Asp residue and facilitated by local hydrophobic environments. The bonds are normally assumed to form when the pilin subunits fold and the three residues involved are brought into proximity. In at least one case, however, it appears that the isopeptide bond does not form until the pilin subunit is incorporated into the pilus; in the N-terminal domain of *B. cereus* BcpA the requisite Lys, Asn and Glu residues are present but do not form an isopeptide bond in the recombinant protein [17], [23].

In *S. pneumoniae* the genes required for pilus formation are located in the 12 kb *rlrA* islet [24] and include three genes encoding proteins with the characteristic LPXTG motif [25]. These correspond to the major pilin RrgB and two ancillary pilin proteins, the tip adhesin RrgA and the cell wall anchor protein RrgC [6]. Also included in the *rlrA* islet are the genes encoding three sortases SrtB, SrtC and SrtD and a positive regulator RlrA [25]. The sortases are assumed to be responsible for incorporation of the various pilin subunits into the growing pilus, as observed in other organisms [9], [11], [12], [26].

As for most of the Gram-positive pili, except for a few that are built from only two components [10], the pneumococcal pilus has a large adhesive pilin at the tip, a polymeric shaft and a smaller cell wall anchor. This pattern of assembly is strongly supported by cryo-EM imaging and immunolabelling of pneumococcal pili [27]. A crystal structure of the adhesin RrgA shows that this 893-residue, four-domain protein has three all-β domains similar to those observed previously in other pilin structures [19]. The C-terminal domain contains an LPXTG motif and is expected to form an isopeptide bond linkage with the N-terminal domain of the major pilin RrgB. No structure is available for the cell wall anchor protein RrgC, but it is predicted to have at least two Ig-like domains which both contain isopeptide bonds as confirmed by MS/MS [28]. Sequence analysis also suggests an additional N-terminal all-β domain that is likely to form the linkage with RrgB.

The 665-residue major pilin RrgB is solely responsible for the extended nature of the pilus, forming its polymeric shaft, which typically comprises 100–200 tandem RrgB molecules. A crystal structure has been determined for a large fragment of RrgB that comprises domains D2 to D4 of this four-domain protein, and can be fitted into the cryo-EM envelope of the pilus shaft [14]. This leaves a portion of the envelope that would be occupied by the missing N-terminal D1 domain, abutting the C-terminus of the next RrgB molecule in the pilus. An NMR solution structure of the isolated N-terminal domain has recently been published [29] but no full-length structure is yet available for RrgB, and several questions remain. Firstly, is the structure of the D2–D4 fragment affected by the absence of D1, and how does D1 dock on to domains D2–D4? This knowledge is required in order to model the complete pilus by docking the atomic structure into the cryo-EM envelope. Secondly, mass spectral analyses indicate that D1 does not contain an internal isopeptide bond even though the requisite amino acid residues are apparently present [28]. Why is this the case, and what does it tell us about the requirements for formation of such bonds?

Here we present the full-length crystal structure of the pneumococcal major pilin RrgB. We show that domains D2–D4 are arranged identically in the full-length structure as they are in the D2–D4 fragment. We further show that absence of an internal isopeptide bond in the D1 domain arises because the β-strand carrying the Asn residue is displaced away from the position required for bond formation. This strand also carries the YPKN pilin motif that provides the Lys residue for the linkage to the next subunit, and we trace the displacement of this strand to the proline in this motif. The structure further suggests that flexibility in the N-terminal domain may be important for pilus assembly, and offers an explanation for the observation that the N-terminal domains of some pilins do not form isopeptide bonds until they are incorporated into the pilus.

## Results

### Structure of RrgB

**Figure 1:**
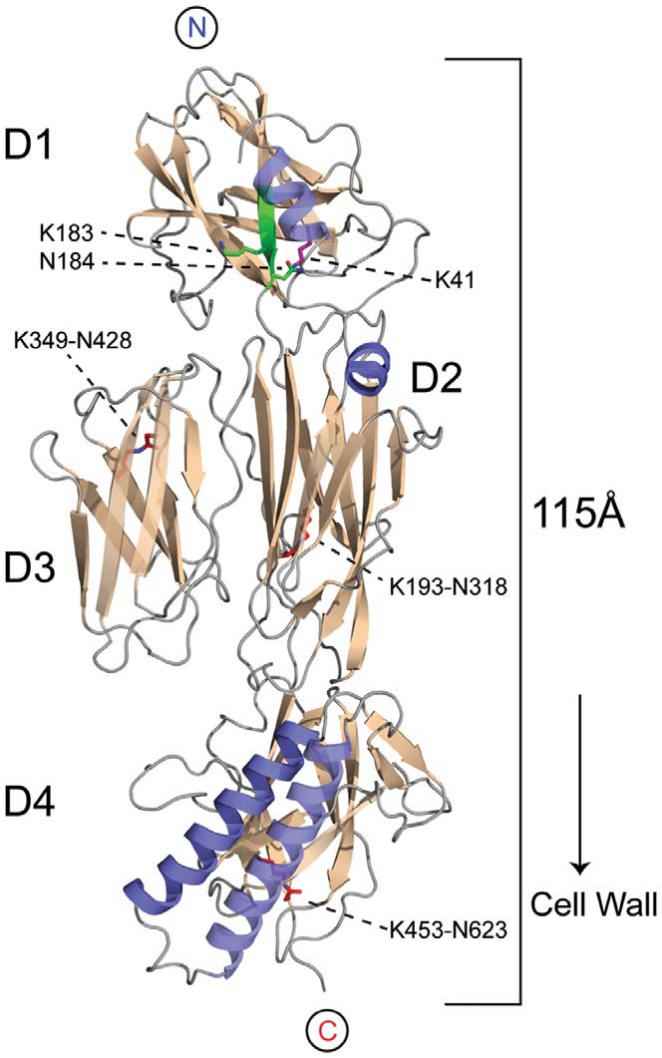
Structure of full-length RrgB. Cartoon representation of the three-dimensional structure of the major pneumococcal pilin RrgB, with the four domains labelled from D1 (N-terminal domain) to D4 (C-terminal domain). β-strands are coloured in wheat and α-helices in blue. In D1, the section of β-strand G that contains the YPKN pilin motif is in green, with the side chains of Lys183 and Asn184 also in green, in stick mode. The side chain of Lys41 from β-strand A in D1 is in magenta, in stick mode. The isopeptide bond crosslinks in domains D2, D3 and D4 are shown in red, in stick mode, and the Lys and Asn residues involved in each case are indicated. The N- and C-termini of the polypeptide chain are also identified.

RrgB is encoded as a 665-residue protein that includes a predicted N-terminal signal peptide of 29 residues [30] and a sortase recognition sequence IPQTG, residues 628–632, that deviates from the classical LPXTG motif. The predicted mature form of RrgB thus comprises residues 30–631. A construct of RrgB comprising residues 30–628 was successfully cloned, expressed and purified. RrgB was crystallized in space group P2_1_2_1_2_1_ and the structure was then solved at 2.8 Å resolution by molecular replacement, using the D2–D4 fragment as a search model. The molecular replacement solution was extended to incorporate electron density corresponding to the D1 domain, with only one small loop between residues 57–61 together with the C-terminal residue 628 not modeled due to inadequate density. This model gives almost complete coverage of the mature form of the protein from signal sequence cleavage site to immediately prior to the sortase cell wall anchor motif. Two essentially identical RrgB molecules (root-mean square (rms) difference in Cα positions 0.61 Å) are present in the asymmetric unit. Full details of the data collection, refinement and structure validation are in Table 1, and the structure of the complete RrgB molecule is shown in Figure 1.

**Table 1:** Data collection and refinement statistics.

Space group	P2_1_2_1_2_1_
Unit cell dimensions (Å, deg)	a = 84.37, b = 107.93, c = 142.45, α = β = γ = 90.0
Data collection§	
Resolution range (Å)	19.88 – 2.80 (2.95 – 2.80)
Wavelength (Å)	0.97946
Total reflections	93755 (14145)
Unique reflections	30964 (4554)
Multiplicity	3.0 (3.1)
Completeness (%)	95.5 (97.5)
<I/σI>	5.6 (2.1)
Rsym (%)	0.20 (0.75)
Refinement statistics§	
Resolution range	19.74 – 2.80 (2.90-2.80)
No. of reflections R_work_+R_free_	30817 (2890)
No. of reflections R_free_	1570 (172)
R_cryst_/R_free_ (%)	21.7/28.1
RMS deviations bond/angles (Å/deg)	0.010/1.32
Average B factor (Å^2^)	29.0

§ Values for outermost resolution shell are given in parentheses.

The crystal structure shows that the D1 domain of RrgB, like the other three domains D2–D4, has an Ig-like fold with the reverse IgG topology, first seen in the repetitive B domains of the collagen-binding adhesin Cna from *Staphylococcus aureus* [31]. This comprises a 4-stranded β-sheet with topology C-B-E-F and a 3-stranded β-sheet G-A-D, in which the first and last strands A and G are parallel. Such domains are typically described as CnaB-type domains and are commonly found in pilins and other cell-surface adhesins [22]. Searches of the Protein Data Bank with Dali [32] and SSM [33] show that the D1 domain of RrgB most closely resembles the N-terminal domain of *C. diphtheriae* SpaA [13] (27% sequence identity, rms difference of 2.5 Å for 97 Cα atoms) and domain 2 (CNA_2_) of *B. cereus* BcpA [17] (17% sequence identity and rms difference of 2.9 Å for 99 Cα atoms). The structure of domains D2, D3 and D4 is unchanged from that seen for the previously-characterized D2–D4 fragment [14], with an rms difference of 0.86 Å for 442 Cα positions. The addition of domain D1 thus does not affect the D2–D4 structure, emphasizing the modular nature of pilin formation.

A notable difference from the other major pilins so far characterized is that in RrgB domains D2 and D3 are packed side-by-side instead of end-on-end. Domain D1 is located above D2, with D3 packed laterally against D2, and D4 located at the base of the monomer, closest to the cell wall (Figure 1). The arrangement along the principal axis is thus D1–D2/D3–D4, giving overall dimensions of approximately 115 Å in length and 40 Å in diameter. D1 makes few contacts with D2, no contacts with D3, and in the crystal structure is offset from the plane formed from D2-D3-D4. The very small number of contacts between D1 and the rest of the structure suggests that in solution there is likely to be some flexibility in the orientation of D1 relative to D2–D4. Contacts are limited to a loop at the base of the helical region of D1, residues 43–45, which packs against two loops at the top of D2, residues 230–233 and 291–294. The idea of increased freedom for D1 is supported by the observation that average main chain temperature factors are higher for D1 (30.7 Å^2^) than for D2–D4 (22.8 Å^2^).

The asymmetric unit contains two molecules that pack side-by-side in antiparallel fashion. In the crystal this generates antiparallel chains of RrgB molecules arranged head-to-tail. While superficially similar to the pilus-like chains seen in the crystals of SpaA and Spy0128 [13], [15], the RrgB chains are generated purely by translation of a monomer, resulting in a pseudo-pilus that lacks any relative rotation of successive pilin subunits. Such a model does not agree in detail with the images obtained by cryoelectron microscopy [27], but it is possible that the limited contacts between D1 and D2 allow rotation of D1 to take on alternative orientations.

### The D1 domain does not contain an isopeptide bond

Mass spectrometry was utilized to measure the intact mass of recombinant RrgB and check for the presence of internal isopeptide bonds; each bond should result in a 17 Da mass difference due to the loss of an NH_3_ molecule, eliminated when the Lys ε-amino group bonds to the Asn carboxyamide group [34]. The measured mass of RrgB of 64778.40 was ∼51 Da less than the theoretical value of 64829.34, indicating the presence of three isopeptide bonds. This was the same as previously observed for domains D2–D4 [14], and is consistent with the formation of isopeptide bonds between Lys193-Asn318, Lys349-Asn428 and Lys453-Asn623, in domains D2, D3 and D4 respectively (Figure 1). Each of these isopeptide bonds is well supported by continuous electron density over the Lys and Asn residues in the present crystal structure.

**Figure 2:**
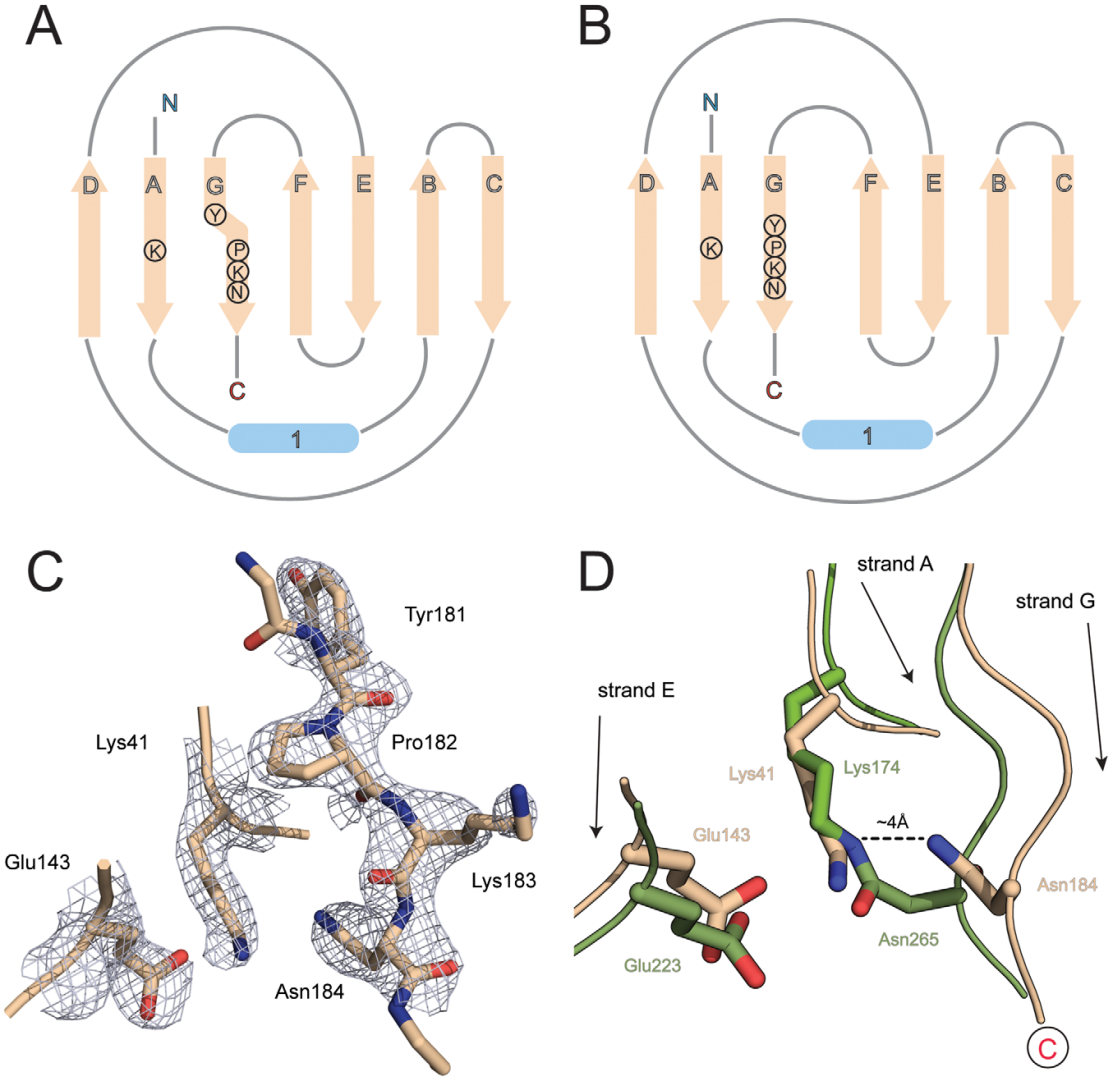
β-sheet topology and isopeptide bond formation. A) Topological representation of domain D1 of RrgB. The final β-strand G carries the YPKN pilin motif residues, which include the lysine used for ligation to the next subunit. In RrgB D1, strand G diverges from the N-terminal strand A at the proline residue of this motif, transferring its hydrogen bonding to strand F. B) In a conventional CnaB domain, which comprises a 3-stranded β-sheet D-A-G and a 4-stranded sheet F-E-B-C and strand G is hydrogen bonded to strand A along its whole length, allowing an isopeptide bond to be formed between a lysine in strand A and an asparagine in strand G. C) Electron density for the YPKN motif (residues 181–184) in β-strand G of domain D1 together with that for the other two residues that could potentially participate in internal isopeptide bond formation, Lys41 and Glu143. The electron density is from a 2Fo – Fc map contoured at 0.38 eÅ^−3^ (1.5σ). D) Superposition of strands A and E of RrgB D1 (wheat) on to the equivalent strands of domain D2 of BcpA from *B. cereus* (green) showing that an isopeptide bond such as that present in BcpA between Lys174 and Asn265 cannot occur in RrgB D1 because the divergence of strand G in RrgB D1 pulls Asn184∼4 Å further away from the lysine (Lys41) in strand A.

Both the electron density (Figure 2) and mass spectral results show that there is no intramolecular isopeptide bond in the D1 domain of our RrgB construct, confirming previous mass spectral results [28] and consistent with the solution structure of this domain [29]. D1 does, however, contain residues that correspond exactly in topological position with those seen in other CnaB-type domains. These are Lys41 on β-strand A of D1, Asn184 on β-strand G (the last strand, leading into the D2 domain) and Glu143 on β-strand E. The strands carrying Lys41 and Asn184 are parallel and spatially adjacent as in other CnaB domains. As in other isopeptide bonding sites the three residues are in a predominantly hydrophobic environment, surrounded by Ile76, Tyr150, Ala163, Phe181 and Pro182.

Structural comparisons with other CnaB-type pilin domains that do contain isopeptide bonds show that the key difference is that in RrgB D1, the strands that carry Lys41 and Asn184 diverge prior to the Lys residue; the last hydrogen bond is between 40 N and 180 O. In contrast, in those CnaB domains that contain an isopeptide bond, these two parallel β-strands remain fully hydrogen bonded to beyond the Asn residue. The consequence of this divergence is that Asn184 is located ∼4 Å away from residues Lys41 and Glu143, preventing bond formation with the Lys side chain (Figure 2). Divergence of strand G from strand A is also associated with a transfer of its hydrogen bonding capability to strand F, the edge strand of the other (4-stranded) β-sheet; residues 183–185 are hydrogen bonded to residues 162–164 of strand F (Figure 2).

### Location of intermolecular isopeptide bond

**Figure 3:**
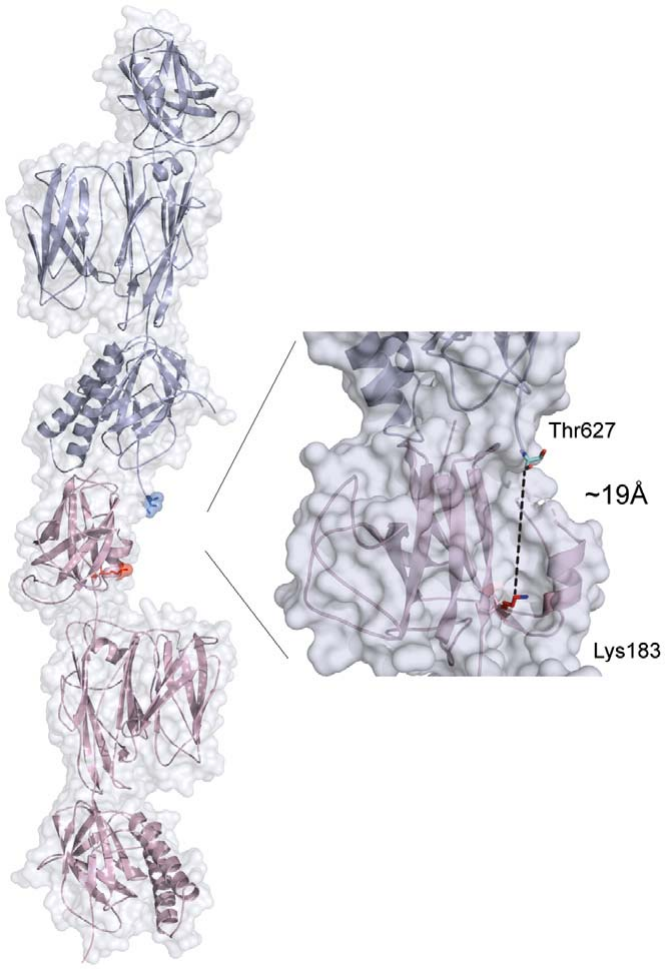
Model for RrgB polymer formation in pneumococcal pili. Cartoon and surface representation of showing the docking of two successive molecules of full-length RrgB. Model is based on the molecular packing observed in crystals of RrgB, but with successive molecules rotated ∼180° in order to conform to the cryo-EM density observed for pneumococcal pili [27]. In this model, the C-terminal residue Thr627 of the crystal structure is ∼19 Å from the ε-amino group of Lys183, a distance that would accommodate the four additional residues 628–631 to the true C-terminus. The enlarged area shows a surface groove into which the missing residues could fit, running from the C-terminal Thr 627 (blue) to Lys183 from domain D1 (red).

Modeling studies based on fitting the D2–D4 structure, together with a modeled D1 domain, into the cryo-EM envelope for the pneumococcal pilus suggested that Lys183, part of the YPKN “pilin motif” might be too far distant from the C-terminus of the preceding monomer for this residue to form the inter-subunit isopeptide linkage [14], although mutagenesis of candidate lysine residues has now shown that Lys183 is indeed the residue involved [29]. The present crystal structure of full-length RrgB provides a useful test of the spatial considerations involved, since although the EM density is not currently available, the asymmetric bulge in RrgB of D3 allows a reasonable approximation of the molecular shape to be compared. If the end-to-end stacking of RrgB molecules in the crystal is taken as a starting model, rotation of one RrgB monomer through ∼180° with respect to the next molecule along the major axis gives an envelope consistent with the EM density and places Lys183∼19 Å from residue 627, which is the last modeled residue of the next molecule (Figure 3). Allowing for the fact that four residues (628–631) are missing to the true C-terminus of the mature protein, this distance should be sufficient to allow a linkage to Lys183. Rotation of D1 also places the helix-turn-helix region from the C-terminal end of one molecule in position to interact with the disordered loop region of the D1 domain of the next, and may stabilize this region in the pilus polymer.

## Discussion

The pili expressed by Gram-positive bacteria are unique examples of covalent biological polymers. They are long and thin, typically 1–5 µm in length but only ∼30–60 nm in width (no more than one molecule wide), but their strength and integrity are maintained by a remarkable sequence of covalent cross-links, both between the individual pilin subunits and within them. In both cases, these cross-links take the form of isopeptide bonds. The intermolecular linkages are catalyzed by the action of the sortase enzymes, which join the C-terminal carboxylate of one subunit to a specific lysine side chain on the next [11]. The internal cross-links, however, arise through autocatalytic, intramolecular reactions that occur spontaneously in the pilin subunits [15], [22].

We focus here on the major pilins, or backbone pilins (BPs), which form the polymeric shaft of the pilus, comprising several hundred tandem BP subunits, arranged like beads on a string. The simplest of these BPs is that from Group A Streptococcus, exemplified by Spy0128 from the M1 SF370 strain of *S. pyogenes*. This protein comprises two Ig-like domains, both of the CnaB-type and each containing an internal Lys-Asn isopeptide bond. Spy0128 also possesses a lysine residue, Lys161, on a surface ω-loop that is extruded from the final strand (G) of its N-terminal domain, and through which it becomes linked to the next molecule in the pilus [15]. The internal isopeptide bonds have been shown to confer thermodynamic stability and protection against proteolysis [21], and to confer remarkable mechanical stability; AFM experiments have shown that the subunits are essentially inextensible, and that this mechanical stability is due to the internal isopeptide bonds [35]. The location of the isopeptide bonds means that a direct line of covalent connections can be traced along the principal axis of the pilus, supporting the view that these provide resistance to the tensile stresses associated with attachment to host cells [15], [36].

Studies on the GAS major pilin Spy0128 first brought to light the paradigm that underlies the structure and stability of Gram-positive pili, although Spy0128 is in several respects atypical of most other major pilins. All the major pilins are modular proteins, made up of multiple Ig-like domains. Spy0128 is unique, however, in having only two Ig-like domains, both CnaB-type and both containing internal isopeptide bonds. In contrast, most of the other major pilins are larger, with SpaA from *C. diphtheriae* and GBS80 from *S. agalactiae* each having three domains [13], [16], and BcpA from *B. cereus* and RrgB from *S. pneumoniae* four domains [14], [17], [27]. GBS80, SpaA and BcpA each also include one domain with a different Ig-like fold, the Dev-IgG fold characteristic of CnaA-type domains, and RrgB has two of its four CnaB domains arranged side-by-side rather than end-on-end.

The larger BPs such as RrgB share several characteristic differences from Spy0128. Firstly, the critical lysine residue used to form the covalent intermolecular linkage that creates the polymeric shaft is present in a conserved YPKN motif, referred to as the pilin motif [37]. No such motif is present in Spy0128 and the lysine is instead located on an ω-loop that is in a similar spatial position but a different sequence context. Secondly, the N-terminal domains in the larger BPs tend to be sensitive to proteolysis and difficult to crystallize; the structures determined for BcpA, GBS80 and RrgB (prior to the present work) all lacked their N-terminal domains [14], [16], [17]. Thirdly, although each of the other domains in the larger BPs has a stabilizing intramolecular isopeptide bond, their N-terminal domains either do not (as in SpaA) or do not form them in the recombinant proteins. Importantly, it has been shown for BcpA that its N-terminal domain has the potential to form an isopeptide bond between Lys37 and Asn163; this bond does not form in the recombinant protein, however, but it is present in native pili, implying that pilus assembly in some way enables the isopeptide bond to form [11]. It is not yet known whether this is true also of the pneumococcal pili, or the pili formed by GBS80.

The present structure enables us to address these issues. RrgB, like BcpA, has an N-terminal domain that contains the appropriate residues for isopeptide bond formation (Lys41, Asn184 and the catalytic Glu143 in RrgB) yet this bond is not present in the recombinant protein. In both RrgB and BcpA the Asn residue of the triad belongs to the YPKN motif, and is immediately preceded by the Lys residue used to form the sortase-mediated intermolecular linkage to the next molecule in the shaft. As we show here, this motif comprises residues 181–184 and is located on the final β-strand (G) of the N-terminal domain (Figure 2A), just prior to the point where it leads into the second domain of the molecule. The side chain of Lys183, which forms the intermolecular linkage, projects into solution between the D2 domain and the disordered loop 57–61 on the D1 domain, whereas those of both Pro182 and Asn184 are directed inwards. The Asn184 side chain is close to that of Lys41, but not close enough to form a Lys-Asn isopeptide bond (Figure 2).

**Figure 4:**
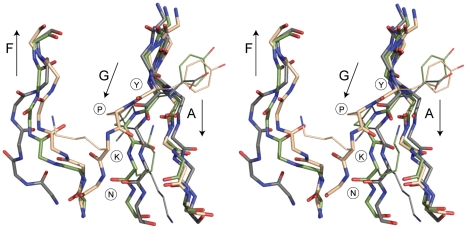
Comparison of β-strand organization in three CnaB-like pilin domains. Stereo figure shows a superposition of strands A, F and G from RrgB D1 (wheat), SpaA D1 (green) and BcpA D2 (grey) showing the divergence of strand G observed in RrgB D1. The positions of the YPKN pilin motif residues are indicated. RrgB D1 and SpaA D1 are the N-terminal domains of their respective proteins and both have a pilin motif (YPKH for SpaA D1); their superposition shows that two possible orientations are possible for strand G in such domains. SpaA D1 has no internal isopeptide bond is it lacks the essential Lys-Glu-Asn triad. In BcpA D2, which has no pilin motif, strands A and G stay together and a Lys-Asn isopeptide bond is formed (shown in Fig. 2).

Structural comparisons of the RrgB D1 domain with other CnaB-type pilin domains show a key difference. In all cases in which a Lys-Asn isopeptide bond is formed, the two parallel β-strands A and G, carrying the Lys and Asn residues respectively, are stitched together with C = O…HN interchain hydrogen bonds along their whole length, from before the Lys to after the Asn. In contrast, in RrgB this hydrogen bonding ceases prior to Lys41, at a position where the NH group of residue 182 should hydrogen bond to the carbonyl oxygen of residue 40. Residue 182 is the proline of the YPKN pilin motif, however, and cannot form such a hydrogen bond. Strand G then diverges away from strand A and hydrogen bonds instead to strand F, the edge strand of the other β-sheet (Figure 4).

What are the implications for isopeptide bond formation? In domains containing a YPKN-type pilin motif the proline residue disrupts the hydrogen bonding between strands A and G. We propose that although this is not in itself sufficient to cause the strands to diverge, it does destabilize the structure, in a similar way to that shown previously for an Ig-like titin domain. In the latter, single-site proline substitutions that disrupt the hydrogen bonding between the A and G strands significantly alter mechanical stability [38]. The structural consequences for pilin domains containing YPKN motifs are difficult to assess, as few structures are available. Nevertheless, from the three pilin structures of this type determined to date, it is apparent that two alternative configurations can be adopted. The A and G strands may diverge, as in the D1 domain of RrgB and the N-terminal domain of the minor pilin GBS52 [39], which has a YPKI pilin motif; in both of these proteins strand G transfers its hydrogen bonding to strand F. Alternatively strands A and G remain together, as in SpaA [13], with other interactions compensating. In those pilins that possess both a YPKN motif and a Lys-Asn-Glu triad, there is evidently a subtle balance. Divergence of strands A and G, as occurs in RrgB D1, pulls the Lys and Asn residues apart, disfavouring isopeptide bond formation in the isolated pilin protein. It may require some external stimulus, such as might be provided by pilus assembly (see later), to overcome the energy barrier, bring strands A and G together, and allow the isopeptide bond to form.

Ig-like domains of this type are evidently sensitive to destabilization, and the interactions between the A and G strands appear to be particularly important. Loss of the isopeptide bond linking strands A and G in the N-domain of Spy0128 causes an ∼30°C reduction in melting temperature [21], and disruption of the interactions between the terminal strands of other Ig-like domains also appears to be destabilizing [38]. The presence, in the N-terminal domains of pilin proteins that incorporate a YPKN pilin motif, of a proline residue in their final β-strand (G) may be one factor that contributes to the observed tendency of such domains to be unstable and prone to proteolysis. The relative lack of contacts between D1 and D2, seen in RrgB, and the high solvent exposure of the linker peptide would further destabilize D1 in this protein. It is evident that, in RrgB, D1 is markedly more mobile than the other domains (average B-factor ∼50% higher than for the other domains). The solution structure of the isolated D1 domain, determined by NMR spectroscopy, also shows that this domain has substantial regions of flexibility [29].

What are the implications for pilus assembly and stabilization? Assembly of the polymeric shaft requires that a pilin-specific sortase cleaves the C-terminal LPXTG motif between Thr and Gly to form a thioester intermediate, then ligates the Thr carboxyl group to a specific lysine on the next subunit. This must require some recognition of the appropriate lysine by the sortase. In all major pilins that have been characterized to date, this lysine is presented by the N-terminal domain, and is usually part of the YPKN pilin motif. Protein-protein recognition often requires some plasticity in one of the partners. As shown by Gentile et al. [29], the D1 domain is the most effective of the domains in RrgB in giving immune protection, possibly because its flexibility allows enhanced antigen-antibody recognition. In the same way, the flexibility of the N-terminal domains of pilin subunits may be functionally important in enabling recognition of the appropriate lysine and docking of the sortase to the pilin surface. The observation that, in BcpA at least, the isopeptide bond in the N-terminal domain is not formed in the recombinant protein but is present in the native pili suggests that it may only be formed after the sortase docks to the pilin. The extra rigidity that would be imposed by an internal isopeptide bond may be counter-productive to correct recognition of the lysine. Importantly, however, the energy associated with the docking of pilin and sortase may result in a repositioning of strand G, which carries the YPKN motif, such that it becomes fully hydrogen bonded to strand A and the internal isopeptide bond forms. These ideas have yet to be tested experimentally, but they do fit all currently-available structural and functional data.

## Materials and Methods

### Cloning, Expression and Purification

*S. pneumoniae* TIGR4 genomic DNA encoding residues 30–628 was amplified by a polymerase chain reaction (PCR) using the following primers, 5′ - GGCAGCGGCGCGGCTGGGACGACAACAACATCT - 3′ and 5′ - GAAAGCTGGCTAGATAGTGATTTTTTTGTTGACTACTT - 3′. Nested PCR (GGGGACAAGTTTGTACAAAAAAGCAGGCTTCGAAAACCTGTATTTTCAGGGCAGCGGCGCG -3′ and 5′-GGGGACCACTTTGTACAAGAAAGCTGGGTG -3′) was used to include a TEV-protease site and permit cloning into Gateway vector pDEST17 (Invitrogen), providing an N-terminal His_6_ tag. This vector was transformed by electroporation into BL21(DE3) chemically competent cells. Cells were incubated in Magnificent Broth (MacConnell Research, San Diego) at 18°C until the OD_600_ reached 0.6, at which point selenomethionine was added to a final concentration of 1 g l^−1^. Protein expression was induced by addition of IPTG to 1 mM. Cells were grown for further 24 h at 18°C before harvesting by centrifugation and storage at −20°C until required. Bacterial cell pellets were resuspended in 50 ml of buffer A (50 mM Tris-HCl pH 8.0, 300 mM NaCl, 25 mM imidazole) and lysed with a French press, after which the soluble fraction was collected by centrifugation. This soluble fraction was loaded on to a 15 ml Ni-NTA column, washed with buffer A until A_280_ reached a baseline value, and then eluted with buffer B (50 mM Tris-HCl pH 8.0, 300 mM NaCl, 250 mM imidazole). The protein was incubated at 4°C with recombinant TEV protease at an approximate ratio of 10∶1. Following cleavage, the protein/TEV mixture was passed through a freshly stripped and recharged Ni-NTA column to remove cleaved His_6_ tags and uncleaved protein. The protein was then concentrated by centrifugation and purified by gel filtration on a Superdex 75 column into crystallization buffer (10 mM Tris-HCl pH 8.0, 50 mM NaCl). Purified RrgB was concentrated to 100 mg ml^−1^ and aliquots stored at −80°C until required.

### Crystallization and Data Collection

Initial crystallization trials were carried out with a Cartesian nanolitre dispensing robot, using sitting drop vapor diffusion in 96-well Intelliplates (ARI, Sunnyvale) and an in-house 480-component screen [40]. These experiments used a reservoir volume of 80 µl, and a drop comprising 100 nl of 100 mg ml^−1^ RrgB solution and 100 nl of precipitant solution. Initial hits from a precipitant comprising 2.0 M ammonium sulfate, 100 mM Tris-HCl pH 8.0 were very small, poor quality crystals that proved difficult to reproduce or optimize. A crystal suitable for data collection was obtained by scaling up the initial conditions into a 24-well hanging drop plate with a reservoir volume of 500 µl and drop comprising 2 µl 75 mg ml^−1^ RrgB solution and 2 µl precipitant solution (1.9 M ammonium sulfate, 0.1 M Tris-HCl pH 8.0, 100 mM ammonium iodide).

A crystal of RrgB was flash-cooled to 100K using paraffin oil as a cryoprotectant [41] and data were collected on beamline MX2 at the Australian Synchrotron (Melbourne), over a total oscillation range of 360° in 0.5° increments. Radiation damage was evident during the collection and only the first 75° of data were deemed suitable for use.

### Structure Solution

Diffraction data were processed using XDS [42], [43] and scaled with SCALA [44], [45]. The data showed distinct anisotropy, and although some data could be seen extending to higher resolution, the data set used for structure solution and refinement was cut off at a resolution of 2.8 Å, where the merging R-factor was 0.75. The crystals were found to have space-group P2_1_2_1_2_1_, with unit cell dimensions a = 84.37, b = 107.93, c = 142.45 Å, α = β = γ = 90.0° and 2 molecules in the asymmetric unit (V_M_ = 2.50 Å^3^/Da, 50.9% solvent content). Radiation damage to the crystals prevented the use of selenomethionine anomalous diffraction for phasing. Instead, the structure was solved by molecular replacement, using PHASER [46], with residues 186–627 from chain A of the D2–D4 fragment of RrgB [14] (PDB entry 2X9W) taken as a search model. The missing residues 33–185, which constitute domain D1, were added manually with COOT [47] with the exception of residues 57–61 which lacked clear electron density. Model refinement was carried out using BUSTER [48] to final values of R_cryst_ = 0.217 and R_free_ = 0.281 (5% of reflections selected randomly for the R_free_ calculation). The final model comprised two molecules of RrgB (590 residues each) with only residues 57–61 and the C-terminal residue 628 missing. Validation with Molprobity [49] showed that 97.1% of residues fell into the most favored regions of the Ramachandran plot. Full details of data collection and refinement statistics are provided in Table 1. Model coordinates and structure factors are available at the PDB under entry 3PRK.

### Mass Spectrometry

All samples were analyzed by infusion-MS using a QSTAR XL Quadrupole Time of Flight mass spectrometer (Applied Biosystems, Foster City). RrgB at a concentration of 100 mg ml^−1^ was diluted 150-fold in 50% acetonitrile, 0.1% formic acid in water. Samples were scanned from m/z 1000–2500 and the resulting multiply charged spectra were deconvoluted using the Bayesian Protein Reconstruct Tool within Analyst QS (Applied Biosystems, Foster City).
